# Supplementary figures and images for: Heparan sulphate binding controls in vivo half-life of the HpARI protein family
Source: eLife. 2024 Nov 8;13:RP99000. doi: 10.7554/eLife.99000 (PMC11548879; doi:10.7554/eLife.99000)

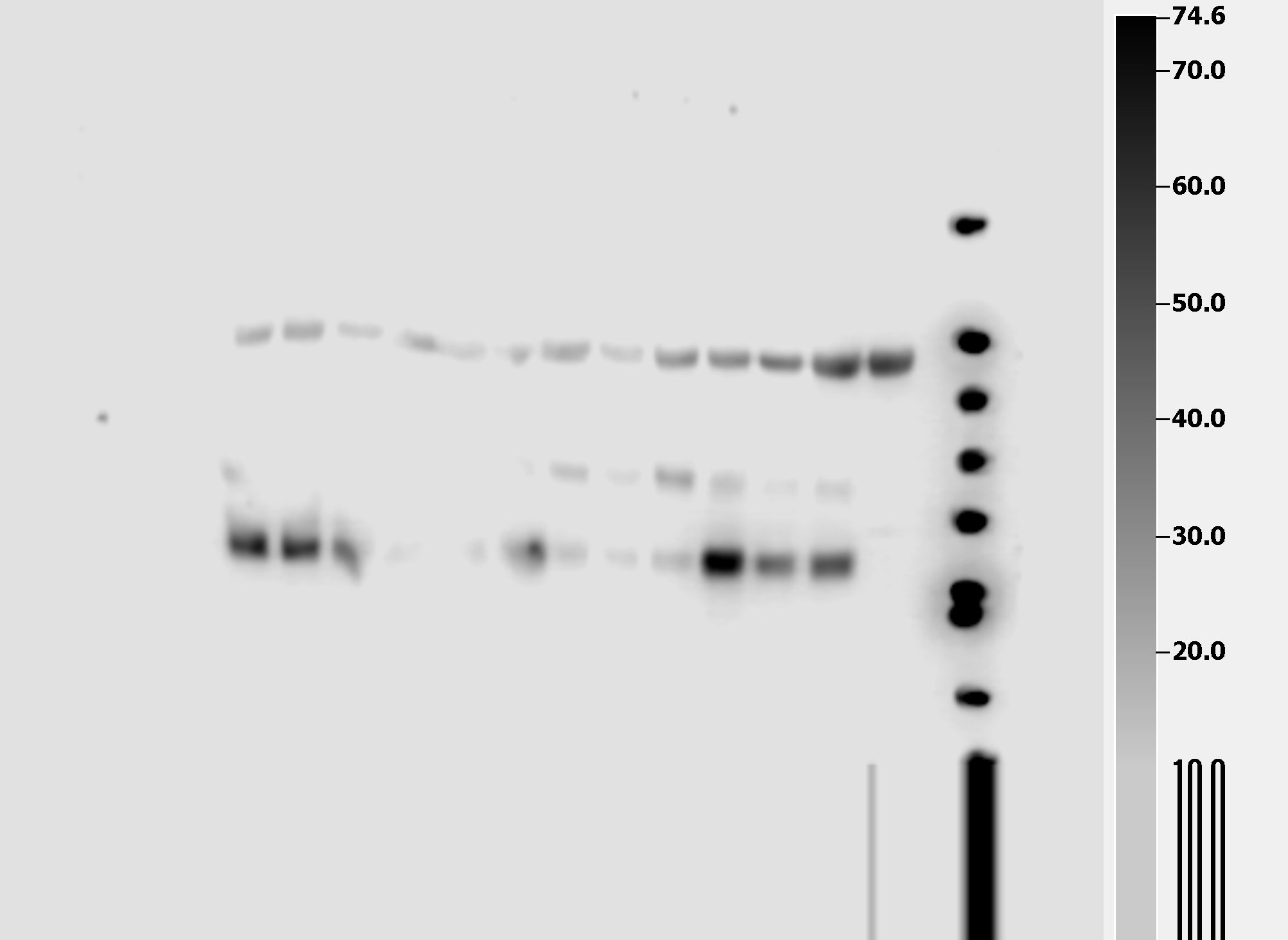

Supplement: Figure 1—source data 1. [file elife-99000-fig1-data1.zip › Source data Fig 1C.tif]

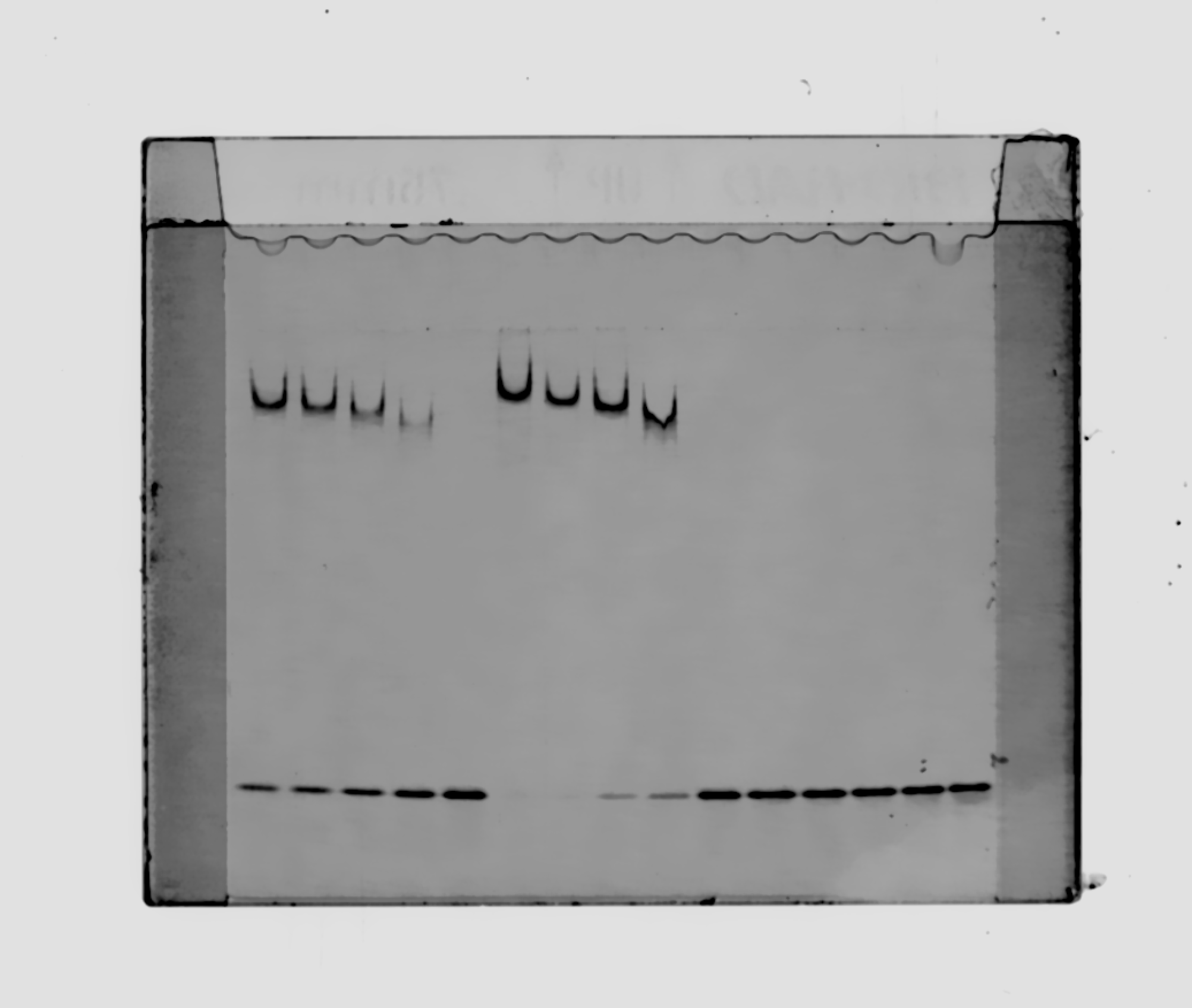

Supplement: Figure 1—source data 1. [file elife-99000-fig1-data1.zip › Source data Fig 1D.tif]

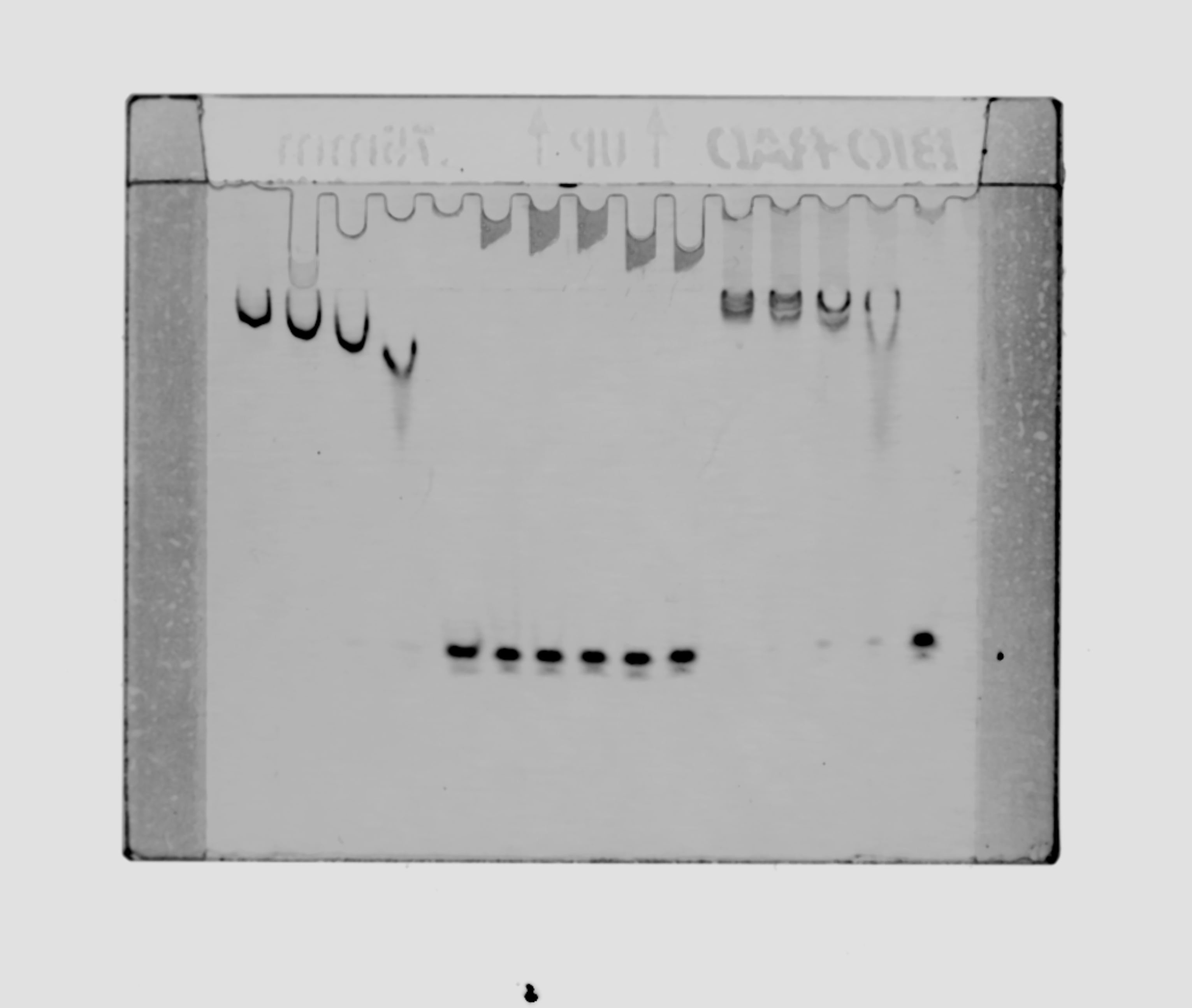

Supplement: Figure 1—source data 1. [file elife-99000-fig1-data1.zip › Source data Fig 1G.tif]

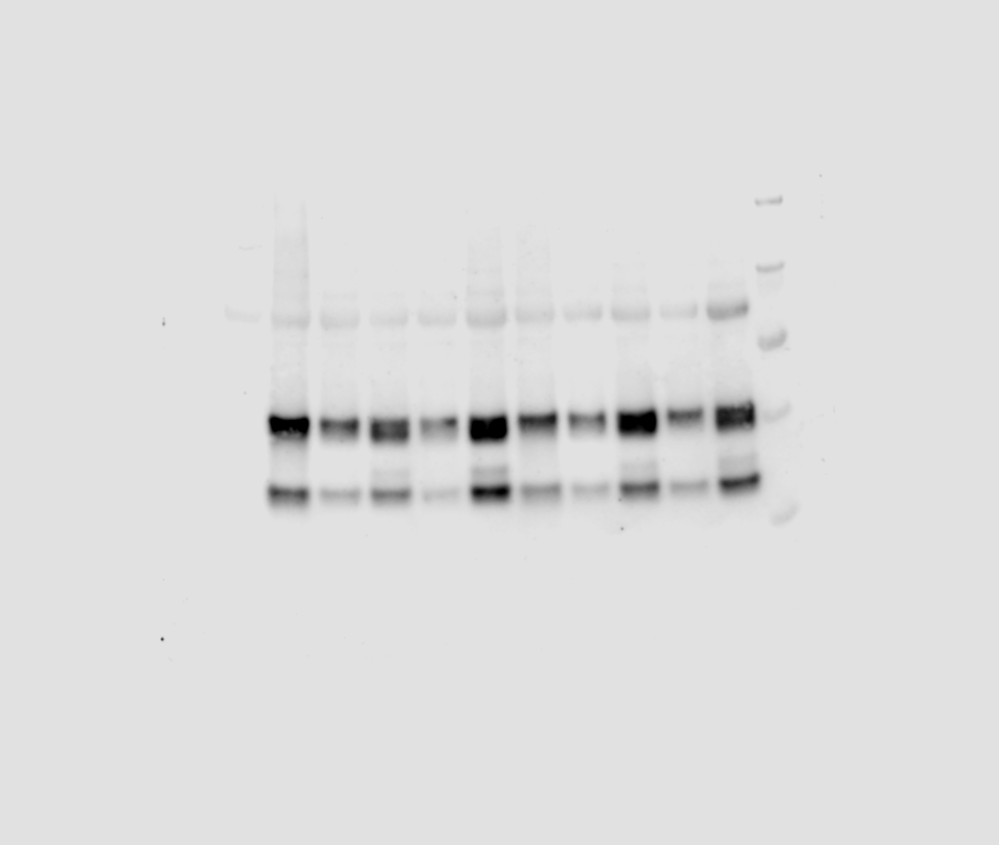

Supplement: Figure 2—source data 1. [file elife-99000-fig2-data1.zip › Source data Fig 2B.tif]

Fig 2B IL-33 western blot

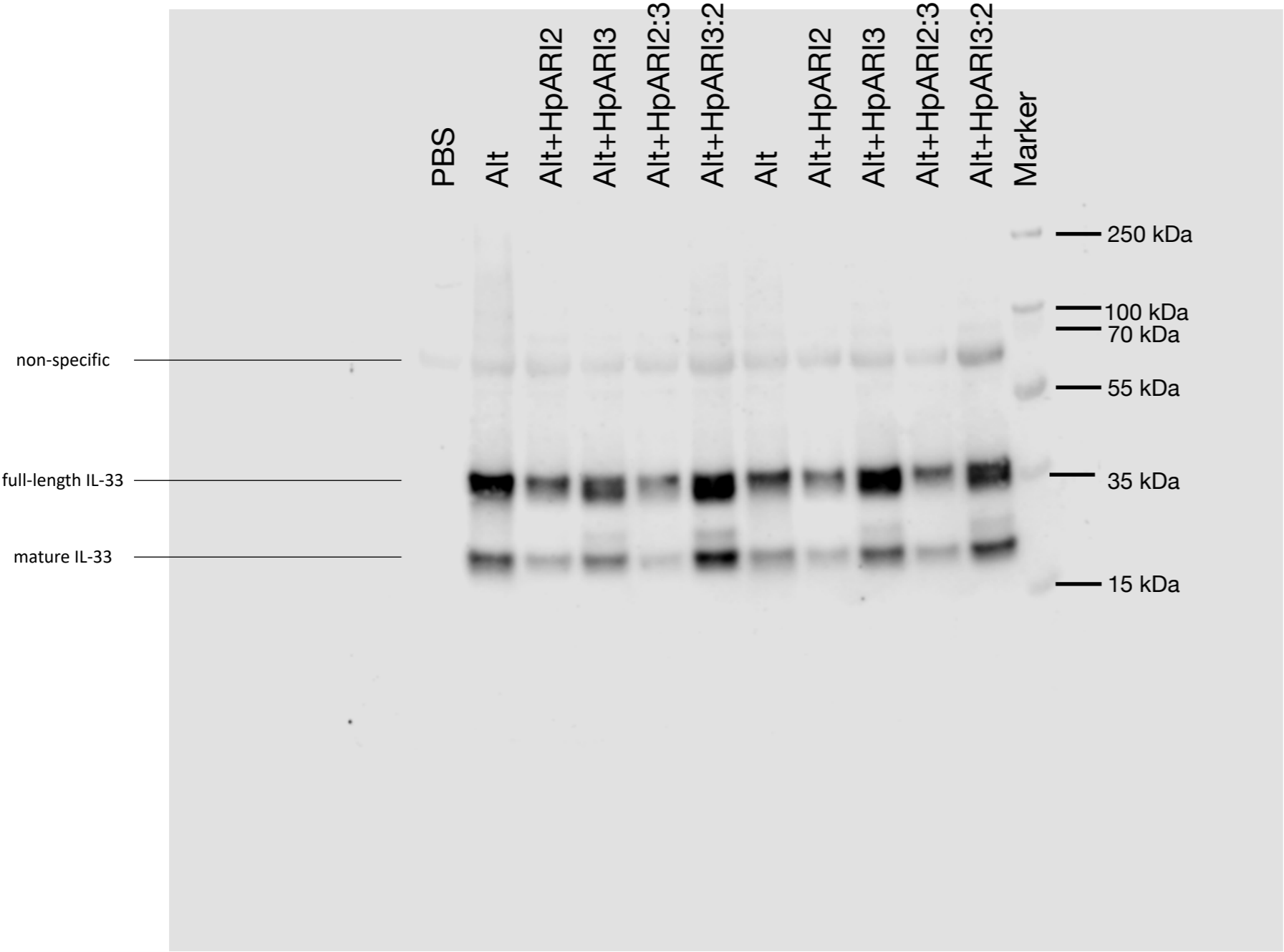

Mature IL-33 band used for densitometry analysis.

Supplement: Figure 2—source data 2. [file elife-99000-fig2-data2.zip › Fig 2B annotated source data.pdf]

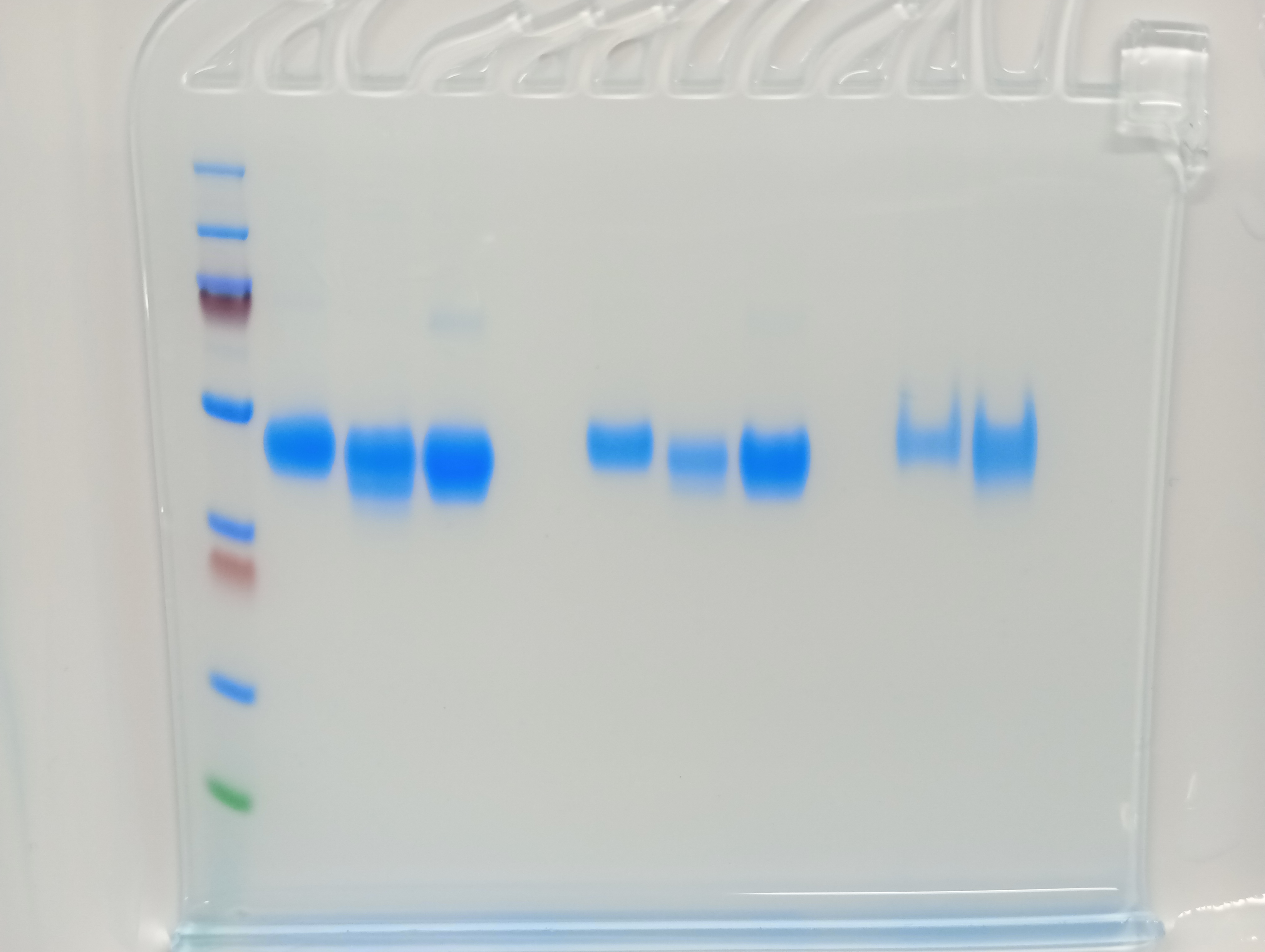

Supplement: Figure 4—source data 1. [file elife-99000-fig4-data1.zip › Source data Fig 4B.jpg]

Fig 4B Coomassie-stained gel

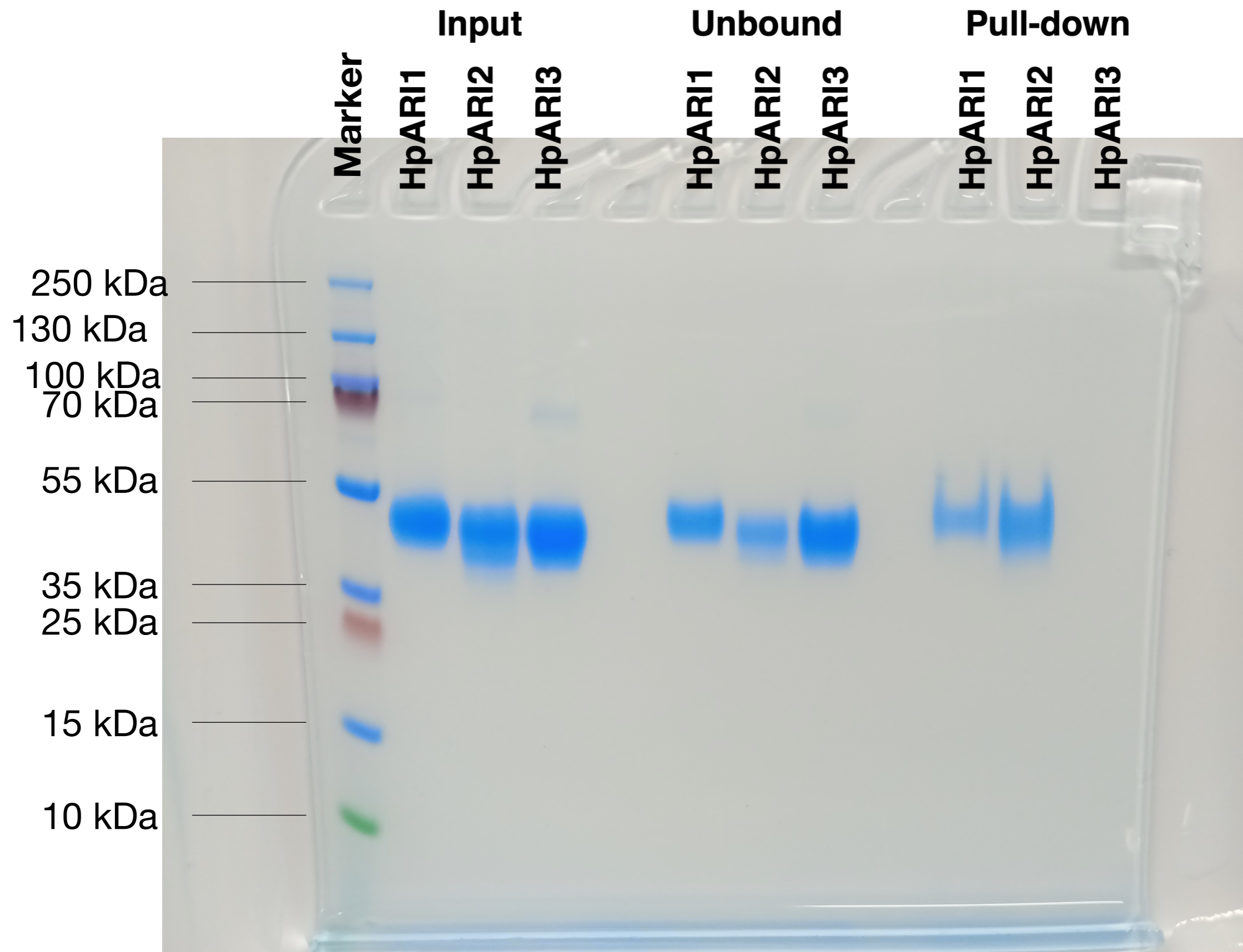

Image made greyscale to show in Fig 4B

Supplement: Figure 4—source data 2. [file elife-99000-fig4-data2.zip › Annotated source data Fig 4.pdf]

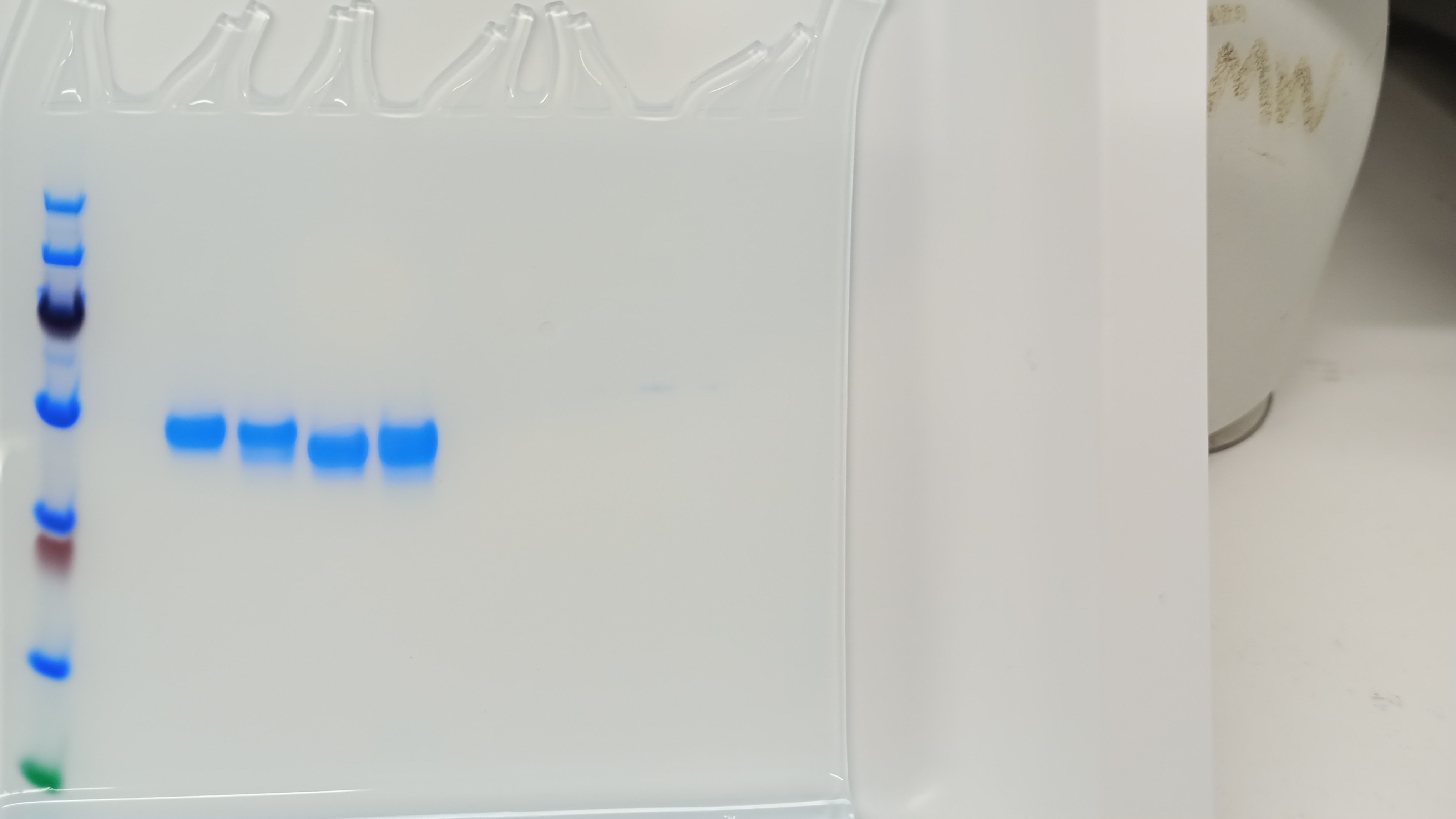

Supplement: Figure 6—source data 1. [file elife-99000-fig6-data1.zip › Source data Fig 6B input.jpg]

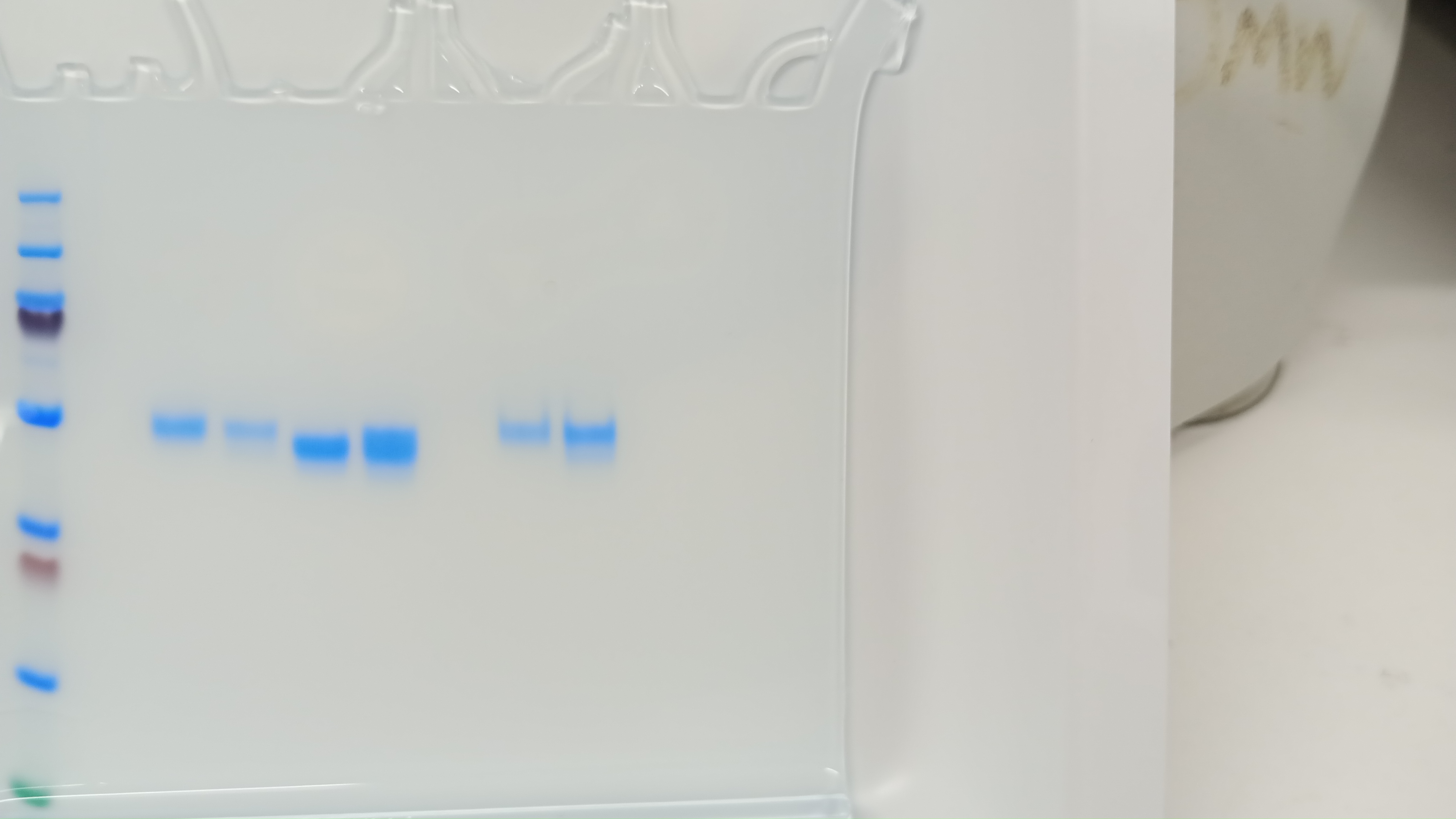

Supplement: Figure 6—source data 1. [file elife-99000-fig6-data1.zip › Source data Fig 6B unbound pulldown.jpg]

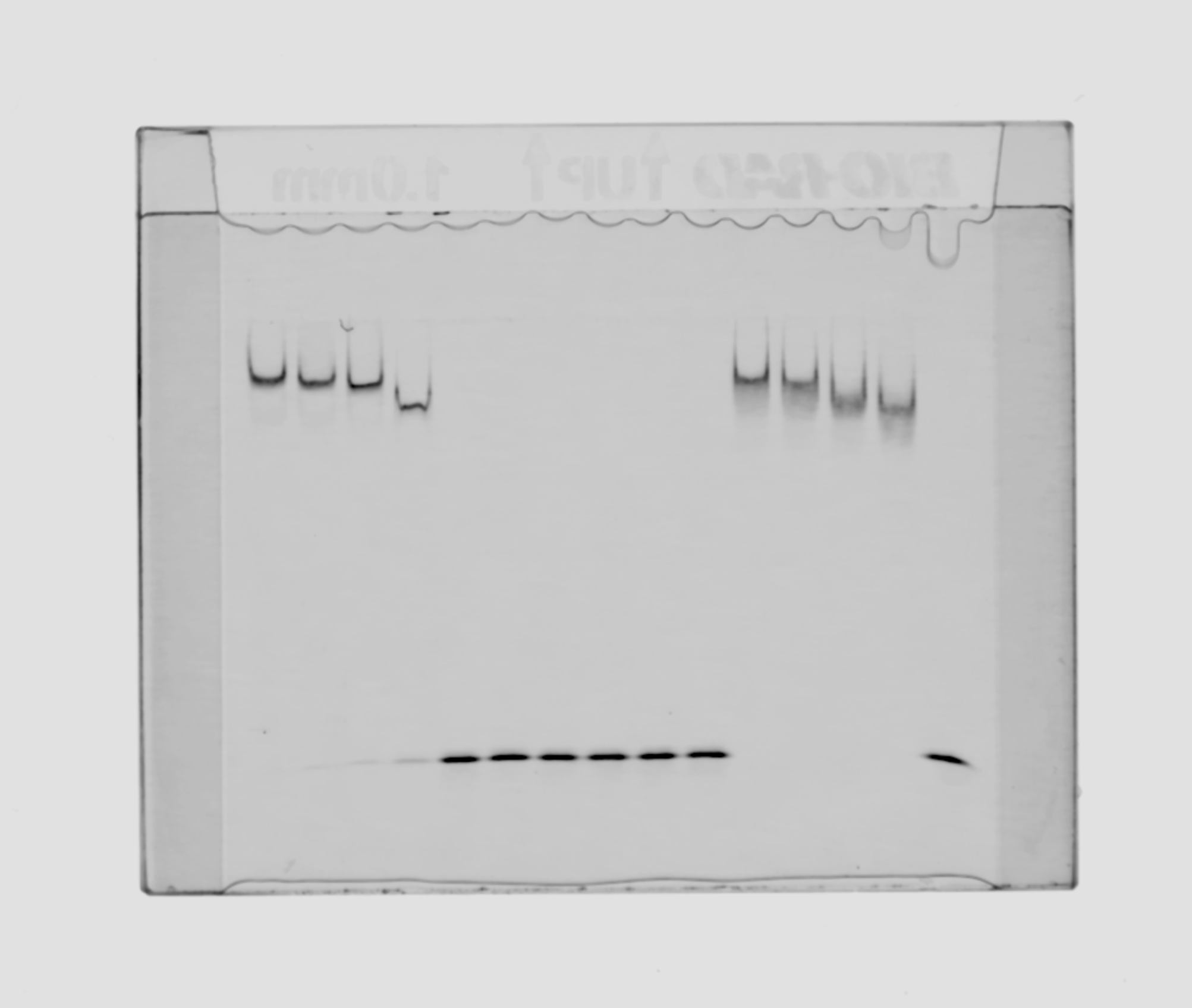

Supplement: Figure 6—source data 1. [file elife-99000-fig6-data1.zip › Source data Fig 6C.tif]

# Fig 6B Coomassie-stained gels

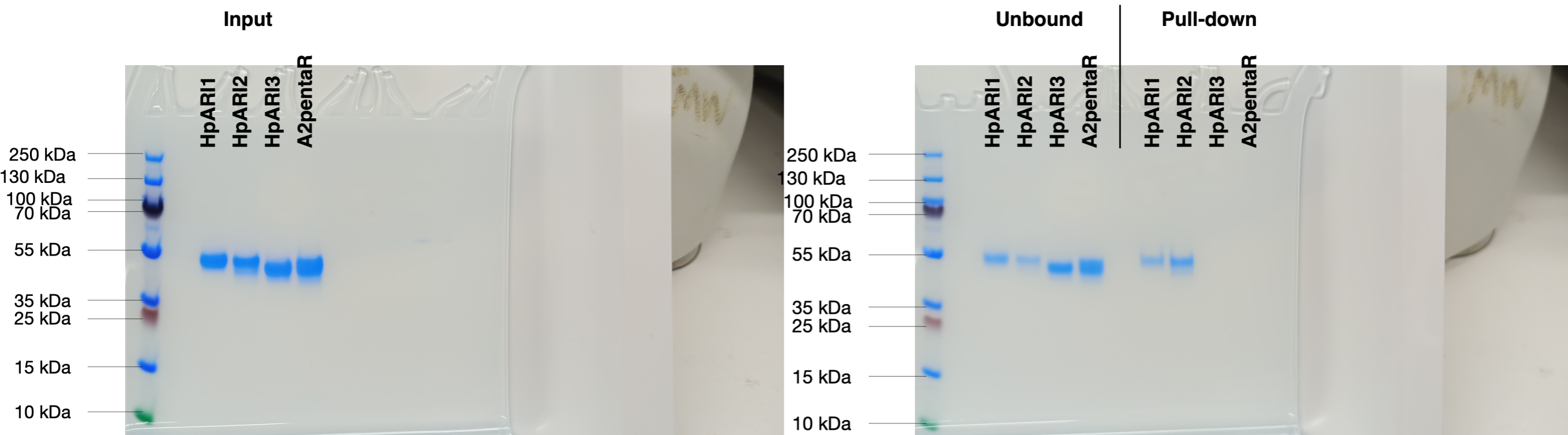

Images made greyscale to show in Fig 6B

# Fig 6C EMSA

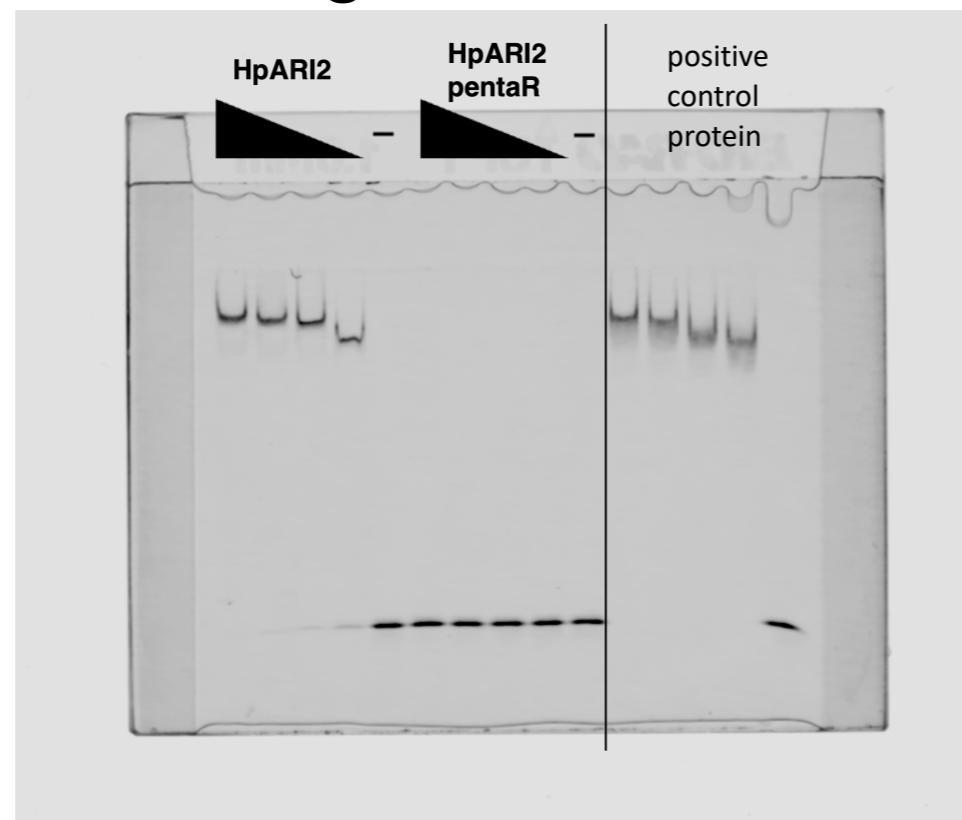

Supplement: Figure 6—source data 2. [file elife-99000-fig6-data2.zip › Annotated source data Fig 6.pdf]
