# Supplementary material for: Heparan sulphate binding controls in vivo half-life of the HpARI protein family
Source: eLife. 2024 Nov 8;13:RP99000. doi: 10.7554/eLife.99000 (PMC11548879; doi:10.7554/eLife.99000)

Fig 1C IL-33 western blot

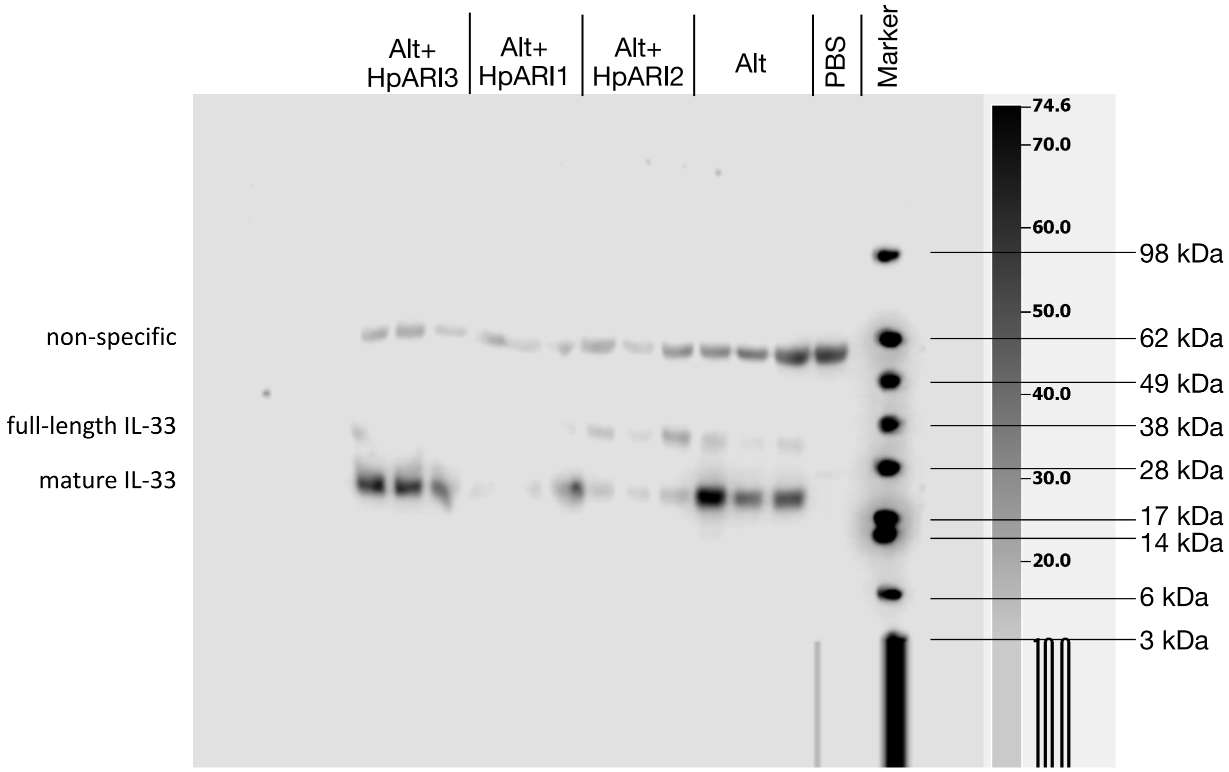

Gel image mirrored to show in Figure 1, Figure Supplement 1.
Artefact at bottom of gel due to image export process and could not be removed.
Mature IL-33 band used for densitometry analysis.

Fig 1D EMSA

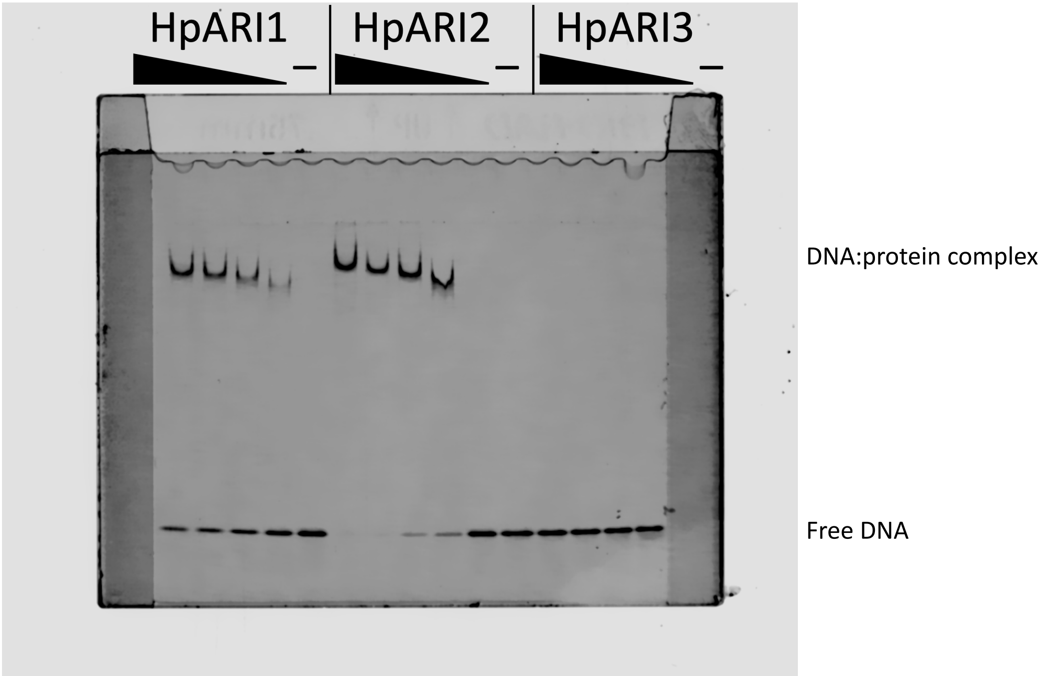

Fig 1G EMSA

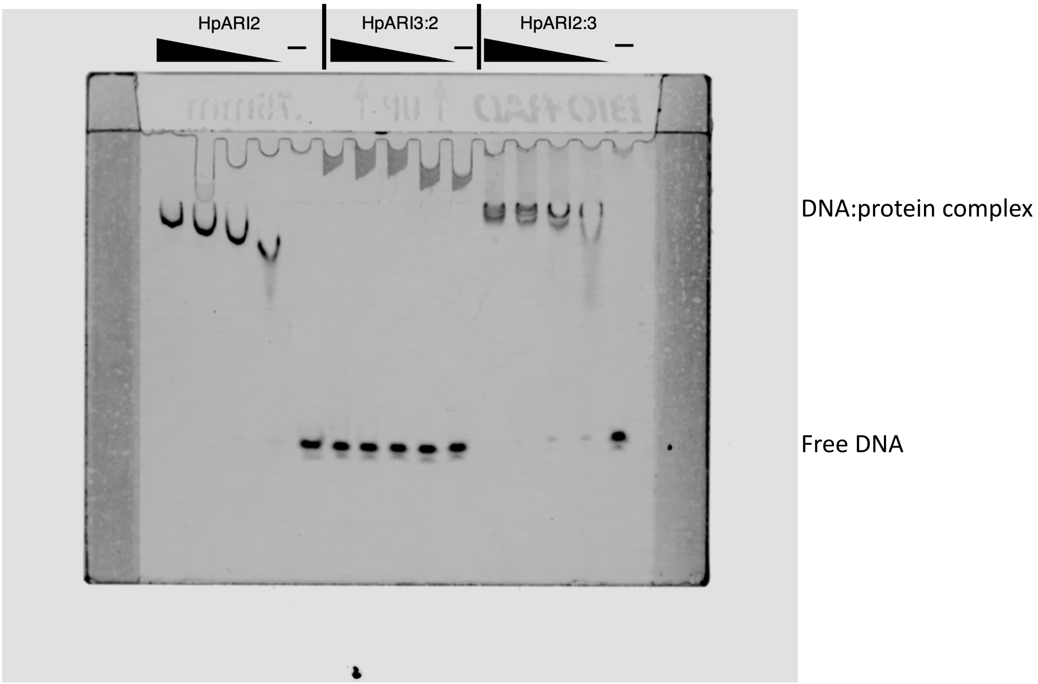

Supplement: Figure 1—source data 2. [file elife-99000-fig1-data2.zip › Annotated source data Fig 1.pdf]
